# Perioperative Management of Dental Surgery Patients Chronically Taking Antithrombotic Medications

**DOI:** 10.3390/ijerph192316151

**Published:** 2022-12-02

**Authors:** Sylwia Wójcik, Katarzyna Mocny-Pachońska, Sophie Bisch-Wójcik, Agnieszka Balicz, Tadeusz Morawiec

**Affiliations:** 1 Department of Dental Surgery, Faculty of Medical Sciences in Zabrze, Medical University of Silesia, Pl. Akademicki 17, 41-902 Bytom, Poland; 2 Department of Conservative Dentistry with Endodontics, Faculty of Medical Science, Medical University of Silesia, Pl. Akademicki 17, 41-902 Bytom, Poland; 3 University Clinical Center, Medical University of Silesia in Katowice, ul. Medyków 14, 40-752 Katowice, Poland

**Keywords:** surgical procedures, dental surgery, antithrombotic, patient management

## Abstract

The development of medicine is based not only on the introduction of new methods of treatment, but also on the use of increasingly effective drugs, including antithrombotic drugs. Drugs that inhibit the activity of platelets (antiplatelet and anti-aggregating drugs) and pharmaceuticals that inhibit the activity of plasma coagulation factors (anticoagulants) are used in antithrombotic therapy. In our daily practice we encounter patients who take chronic antiplatelet or anticoagulant drugs. However, more and more often we are dealing with patients who are treated with two antiplatelet drugs, an antiplatelet and an anticoagulant or even undergoing triple antithrombotic therapy. When preparing the patient for invasive craniofacial procedures, it should be assessed whether the temporary discontinuation of antithrombotic treatment due to the fear of excessive perioperative bleeding is justified and will not result in life-threatening thromboembolic complications. The authors discuss in detail the medications used in modern antithrombotic treatment and present a perioperative management procedure with a patient who takes l4 z of these medications chronically.

## 1. Introduction

Development in the medical field is not only about introducing new treatments, but also about increasing the effectiveness of the types of drugs already in use, including antithrombotics. Antithrombotic therapy involves the use of drugs that inhibit platelet activity (antiplatelet drugs, antiaggregants) and those that inhibit the activity of plasma clotting factors (anticoagulants) [1].

The purpose of this study is to present the management regimen for patients taking chronic anticoagulants and to present the most commonly used drugs in this treatment.

In everyday practice, we encounter patients who chronically take antiplatelet or anticoagulant drugs. More and more often, however, we are dealing with patients who receive combination therapy with two antiplatelet agents, an antiplatelet and an anticoagulant, or triple antithrombotic therapy. When preparing a patient for invasive craniofacial surgery, it should be evaluated whether temporary discontinuation of anticoagulant therapy, due to fear of excessive perioperative bleeding, is justified and will not cause life-threatening thromboembolic complications. The risk and severity of local bleeding can be assessed using the International Society of Thrombosis and Haemostasis questionnaire, which distinguishes three types of bleeding: minor bleeding, major bleeding and severe life-threatening bleeding [1,2,3].

Based on these guidelines, craniofacial procedures can be divided into those with a low, intermediate, and high risk of bleeding [3].

Dental procedures associated with a low risk of bleeding include: opening the pulp chamber, bleeding from the gingival tissue, dental grinding for prosthetic procedures, supragingival scaling with sandblasting, simple extraction of individual teeth, laser removal of fibrous folds, placement of individual dental implants using the flapless technique and laser removal of mucosal lesions (papillomas, hemangiomas) [1,2,3].

During subgingival scaling, procedures in the field of periodontal plastic surgery (gingival augmentation with alloplastic or xenogenic materials), surgical crown lengthening, tooth extraction with flap mobilization and suturing (surgical removal of wisdom teeth, resection, hemisection, radectomy), scalpel excision of fibrous folds, extraction of an alveolar cyst, removal of a small tumor, sample collection for histopathological examination, placement of several dental implants with the creation of a flap, maxillary sinus floor elevation using the closed method, moderate perioperative bleeding may occur [2,3,4].

Procedures during which major bleeding may occur are: Procedures in the field of periodontal surgery (gingival augmentation with autogenous soft tissue grafts from the palatal mucosa), simultaneous placement of more than 6 implants with the creation of flaps, bilateral elevation of the maxillary sinus floor with the creation of a lateral window, miniplate osteosynthesis of the craniofacial bones, reconstructive procedures within the orbit, resection of benign and malignant tumors within the craniofacial region together with reconstructive procedures and orthognathic procedures [1,2].

When deciding to temporarily discontinue antithrombotics, we must consider the increasing risk of thromboembolism and its life-threatening consequences for the patient.

Patients with implanted prosthetic valves regardless of the time since the implantation, patients with implanted biological heart valves or after mitral valve surgery (up to three months after surgery), patients with valvular and non-valvular atrial fibrillation and venous thromboembolism (acute thromboembolic event within the last three months, recurrent thromboembolism, severe thrombophilia) are at high risk of thromboembolic complications after discontinuation of antithrombotics. Therefore, antithrombotic therapy should not be discontinued before surgery. In doubtful cases, consultation with the attending physician is necessary [1,3].

**Figure 1:**
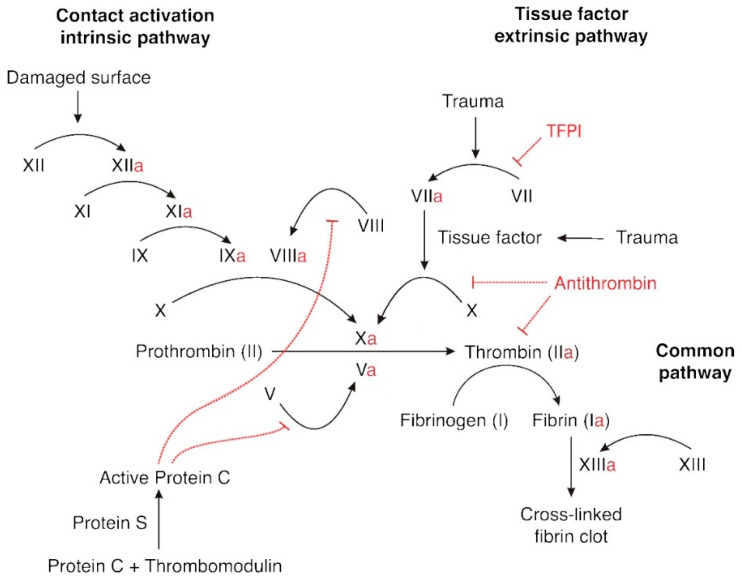
The figure shows a scheme of a coagulation cascade.

During surgical procedures, the continuity of bodily tissues is interrupted. The organism’s response is to accumulate platelets at the site of injury and form a platelet plug. At the same time, the blood clotting cascade begins, involving plasma clotting factors as well as platelets and proteins from the membranes of injured cells (tissue factor). The blood clotting cascade can occur through two pathways. Tissue damage leads to activation of the extrinsic pathway. In contrast, damage to the blood vessel wall activates the intrinsic pathway. In each of these pathways, different clotting factors are activated until they combine into a common pathway, which leads to the conversion of soluble fibrinogen found in plasma, into insoluble threads of fibrin (Figure 1) [5].

Drugs used in antithrombotic therapy work at different stages of the clotting process (Table 1).

## 2. Drugs Used in Antiplatelet Therapy

Acetylsalicylic acid is one of the non-steroidal anti-inflammatory drugs. It has anti-inflammatory, antipyretic and analgesic properties. It irreversibly inhibits the arachidonic acid cyclooxygenase, which leads to a decrease in thromboxane A2 synthesis which irreversibly inhibits platelet aggregation. The therapeutic effect begins 60 min after oral administration. The main indications for the use of ASA are: stable coronary artery disease, ACS (acute coronary syndrome), past stroke or transient cerebral ischemia and atherosclerosis of peripheral arteries. Patients chronically take ASA in doses ranging from 75–100 mg/d p.o. Once the drug is discontinued, the antiplatelet effect persists for 5 days [1,3,6].

Clopridogrel, is a thienopyridine derivative. After oral administration, it achieves its peak effect after 60 min. In blood it irreversibly reacts with P2Y12 receptors, which are located on the surface of platelets and thus inhibits their aggregation. Clopridogrel is used in patients with stable coronary artery disease who have undergone elective angioplasty, patients with ST-elevation myocardial infarction (STEMI), non-ST-elevation myocardial infarction (NSTEMI) as well as patients with unstable angina. The saturation dose is 600 mg [7,8].

Prasugrel (Effient) derives its anticoagulant effect from its permanent binding to the P2Y12 receptor, which is located on the surface of platelets. After oral administration, the onset of action of the drug occurs after 15–30 min. It is used in patients with STEMI, NSTEMI and patients with unstable angina. The commonly used dose is 60 mg [7,8].

Ticagrelor (Briliqe) also belongs to antiplatelet drugs that inhibit platelet aggregation by binding, like the previous two drugs, to the P2Y12 receptor on the surface of platelets. The onset of action occurs 30 min after oral intake. Treatment with 180 mgs of ticagrelor is used in patients with unstable angina, STEMI and NSTEMI [3,6,7,8].

Glycoprotein IIb/IIa inhibitors interfere with platelet cross-linking and thrombus formation by blocking the GP IIb/IIa receptor. This group of drugs includes: Abciximab, Eptifibatide, and Tirofiban. They are administered intravenously in patients undergoing percutaneous coronary interventions (PCI) and in acute coronary syndromes (ACS) [9,10].

Treatment with these antiplatelet agents, especially with dual antiplatelet therapy, is associated with hemorrhagic complications. In addition, some patients may develop tolerance to ASA and clopidogrel. Therefore, other anti-aggregation drugs might be used. Those include: TxA2 synthetase inhibitors (primagrel, dazoxiben, ozagrel, camonagrel), prostacyclin PG12 and its stable analogues (iloprost, cicaprost, taprosten), prostaglandin E1 PGE1 and its analogue misoprostol, eicosapentaenoic acid-EPA (Maxepa) [11].

## 3. Anticoagulants

Anticoagulants are divided into three pharmacological groups: vitamin K antagonists, anticoagulants that are not vitamin K antagonists and heparins and pentasaccharides [12].

The vitamin K antagonists (VKAs), Acenocumarol and Warfarin, are coumarin derivatives. The mechanism of action is based on the inhibition of vitamin K. As a result, deficient blood clotting factors (II, VII, IX, X) are synthetized by the liver. The onset of action occurs after 2–4 days, since these drugs do not act on already produced clotting factors [12].

Acenocumarol has a shorter duration of action which lasts about 2–3 days, while warfarin has a longer duration of action and the anticoagulant effect lasts up to 5 days after its withdrawal. VKAs are indicated for the prevention of embolic complications in patients with atrial fibrillation (AF), patients with mechanical valves and for the treatment of venous thromboembolism (VTE). The dosage of VKAs is individually adjusted for each patient, and the effectiveness of anticoagulant treatment is monitored by the international normalized ratio (INR), which should be between 2–3.5 in patients treated with VKAs. If the patients INR has significant fluctuations, a careful analysis of the prescribed medications and diet should be performed, as VKAs interact with various drugs and substances that reduce or increase their anticoagulant effect [1,12,13].

Oral anticoagulants, non-vitamin K antagonists (NOACs) are divided into two pharmacological groups [12,14].

The first group are direct thrombin inhibitors (dabigatran), the second includes drugs that are direct inhibitors of activated factor X (rivaroxaban, apixaban and endoxaban). NOACs are used in anticoagulant therapy in patients with nonvalvular atrial fibrillation, prevention and treatment of VTE as well as patients undergoing hip and knee replacement. In patients treated with these drugs, there is no need to monitor the anticoagulant effect, nor is there a widely available and standardized laboratory method that would allow us to monitor the anticoagulant effect. In emergencies, the drug concentration in the patient’s blood is measured [14,15,16].

The direct thrombin inhibitor dabigatran (Pradaxa), specifically and reversibly blocks thrombin. Two doses are prescribed to patients (2 × 110 mg or 2 × 250 mg). The effect of the drug appears 2–3 h after administration and persists for 24 h. Dabigatran is eliminated mainly by the kidneys and its use and duration of anticoagulating action is dependent on renal function [14,16].

Direct factor X inhibitors are: rivaroxaban (Xarelto), apixaban (Eliquis) and endoxaban (Lixiana). They work by inhibiting active factor X. The effect occurs after 2–4 h, depending on the type of drug, and lasts for about 12 h [1,14,15,16,17,18].

Another group of drugs used in anticoagulant therapy are heparins and pentasaccharides [19,20].

Two types of heparins are used in clinical practice: unfractionated heparins (UFH) and low molecular weight heparins (LMWH) [19,20].

Unfractionated heparins are not absorbed from the gastrointestinal tract and therefore they are used in intravenous infusions or subcutaneous injections (subcutaneous administration has a bioavailability of 30%). UFH administered intravenously is used in the initial treatment of VTE, ACS, acute peripheral arterial embolism, intracardiac thrombosis, prosthetic valve thrombosis and intravascular catheter thrombosis. UFH are used for in-hospital treatment of patients with AF or prosthetic heart valves. The mechanism of action is based on promoting the thrombin-antithrombin binding leading to thrombin (factor II) inactivation. A lesser inhibitory effect is observed on factors: Xa, IXa, XIa and XIIa. When administered intravenously, the anticoagulant effect occurs immediately after injection. The drugs residence time depends on the administered dose and may range from 1–5 h. When injected subcutaneously, the anticoagulant effect occurs after 2–4 h [1,15,19,20].

Low molecular weight heparins (LMWH) are more potent in inhibiting factor Xa activity with a lesser effect on the thrombin-antithrombin reaction. Individual LMWHs have different inhibitory effects on factor II and factor X, which results in a different anticoagulant effect for a given drug. Low molecular weight heparins are mainly used subcutaneously. After administration, the maximum anticoagulant effect occurs 2–3 h after administration and lasts up to 24 h [19,20].

Pentasaccharides (fondaparinux, idraparinux and idrabiotaparinux), selectively inhibit active factor X. Their mechanism of action involves reversible binding to the antithrombin molecule, which causes changes in its conformation and increases its ability to bind and inactivate factor X. The biological half-life is about 15 h, so they can be used once a day. They are not metabolized in the body and are excreted entirely through the kidneys. Pentasaccharide drugs are administered subcutaneously or less frequently intravenously. They are used in the treatment of VTE, after major surgery of the lower limbs and abdominal region. They are also used after myocardial infarction, coronary artery disease and coronary artery bypass grafting (CABG). Drugs in this group have a more predictable effect and a more convenient dosing regimen compared to conventional treatment. Pentasaccharides also do not require laboratory monitoring and have only few interactions with other drugs or food. Potential limitations of pentasaccharide treatment include potential harm in patients with renal failure and the need for intravenous or subcutaneous administration [1,21].

## 4. Perioperative Antithrombotic Therapy Management

When proceeding to procedures with a low and intermediate risk of local bleeding, antiplatelet therapy should not be interrupted, even for patients who are taking two antiaggregants. In patients taking one antiplatelet agent, it should not be discontinued even for major maxillofacial surgery procedures. Scheduled procedures with a high risk of bleeding, in patients treated with two antiplatelet agents, should be deferred until the end of treatment, approximately 12 months. In urgent cases, consultation with the attending physician is necessary. It is possible, to discontinue one antiplatelet drug. According to their duration of action, clopidogrel and ticagrelor should be withdrawn 5 days before the procedure and prasugrel 7 days. After the procedure, a saturating dose of the discontinued drug should be given within 24–72 h. In emergency situations, it is best to perform the procedure in a hospital with an interventional cardiology unit (Table 2). If stent thrombosis recurs, a coronary intervention will be available [1,2,3,6,8,22,23,24,25].

In patients who are continuously taking vitamin K antagonists, anticoagulant therapy should not be discontinued for procedures with low to moderate bleeding risk. The INR should be tested 24 h before the procedure and should be below 3. If the INR value is above 3, the procedure should be postponed until the clotting time normalizes. In case of a high risk of bleeding, the attending physician should be consulted before performing the procedure. Anticoagulant therapy should not be discontinued in these patients. In the perioperative period, the anticoagulation effect should be temporarily reduced to an INR value of 2.0–2.5. In patients at high risk of thromboembolism, after consultation with the attending physician, bridging anticoagulation therapy should be used in the period preceding surgery with a high risk of bleeding [15,25,26].

Acenocumarol should be is discontinued 3 days and warfarin 5 days before the scheduled surgery. Intravenous UFH therapy or subcutaneous LMWH therapy should be initiated at full dosage when the INR value decreases below 2. UFH requires inpatient treatment and is preferred in patients with prosthetic heart valves. In other cases, LMWH is used as bridging therapy and should be discontinued 12 h before surgery. UFH administration should be discontinued 6 h before surgery. In the postoperative period, anticoagulant therapy should be given with full-dose UFH or LMWH, simultaneously with acenocumarol or warfarin. Heparin administration should be discontinued when the INR reaches a therapeutic value for two consecutive days. In cases of emergency surgery in patients on VKA with a high risk of local bleeding, it is recommended to transfuse fresh frozen plasma (FFP) or prothrombin complex concentrate together with low doses of vitamin K (2.5–5.0 mg) intravenously or orally as well as red cell concentrate transfusion when the hemoglobin level falls below 7 g/dL, in order to reduce the anticoagulant effect (Table 3) [19,23,24,25,26].

There is no need for bridging therapy in patients taking continuous NOAC anticoagulants. In every case, the drug should be discontinued before scheduled surgery. The duration of NOAC discontinuation depends on the type of drug, renal function (as determined by creatinine clearance GFR) and the risk of local bleeding associated with the procedure [15,16,17].

If normal hemostasis is achieved after surgery, return to NOACs is recommended, on the day of the procedure (Table 4) [18].

In cases of immediate surgery, drug-specific antidotes may be considered. For Xarelto—andexanet alfa and for dabigatran—idarucizumab. Prothrombin complex concentrate (PCC) or activated PCC may also be considered. In all patients requiring surgery for urgent indications, baseline coagulation assays should be performed. However, their interpretation is not explicit and the most reliable test is to determine the blood concentration of a particular drug [15,23,24].

In cases when hemostasis does not normalize in the postoperative period, heparin use is recommended. Returning to NOACs, in patients temporarily taking heparins is as follows. The NOAC dose the patient was taking prior to surgery, should be administered 2 h before or during the time normally dedicated to the heparin injection [2,18,23,24].

## 5. Patients on Combination Therapy: VKA/NOAC, VKA/ASA, VKA/Clopidogrel

In procedures with a low and moderate risk of bleeding, during VKA/NOAC treatment we determine the INR 24 h before the procedure, its value should be below 3. NOACs should be discontinued according to the table presented above. If these patients are scheduled for surgery with a high risk of bleeding, bridging anticoagulation therapy should be administered after consultation with the attending physician. The second option is lowering the intensity of anticoagulation therapy to an INR of 2.0–2.5, and discontinuing NOACs according to the scheme (Table 5) [23,24,25,26].

In patients taking simultaneously VKA and ASA or VKA and clopidogrel in case of minor or moderate bleeding risk, we determine the INR value 24 h prior to surgery. Interruption of ASA and clopidogrel treatment is not recommended. In case of major bleeding risk after consultation with the attending physician, ASA and clopidogrel should not be discontinued while VKA therapy should be managed as described in the previous paragraph (Table 6) [25,26,27].

Patients with indications for antithrombotic therapy due to AF, are more and more frequently receiving triple therapy after a coronary intervention consisting of VKA/NOAC with ASA and clopidogrel [1,23,24,25,26].

All scheduled procedures, in patients on triple antithrombotic therapy should be postponed and in urgent cases consultation with the attending physician is necessary.

When performing surgical procedures in patients continuously taking antithrombotic therapy, it is also important to remember to use appropriate techniques and procedures designed specifically to minimize their invasiveness. Particular attention should be given to the use of tools and devices that reduce the severity of perioperative bleeding as well as to achieving and maintaining normal homeostasis [1,22,25,26,27].

## 6. Conclusions

Patients who chronically take antithrombotic drugs can safely undergo dental and maxillofacial surgery procedures.

Before any procedure, the thromboembolic risk associated with antithrombotic drug(s) withdrawal and the severity of perioperative bleeding should be assessed.

When performing procedures with a low risk of perioperative bleeding, do not discontinue antithrombotic treatment, even in patients taking combination therapy of two antithrombotic drugs. If patients are taking NOACs, it is necessary to be very precise about the individual doses of these drugs.

When planning procedures with moderate to high risk of bleeding and in emergency cases, it is necessary to consult the modification of antithrombotic treatment with a cardiologist or neurologist.

Patients with atrial fibrillation (AF) or after coronary intervention receive triple anticoagulant therapy. In these patients, elective procedures, should be postponed, and in case of emergency, the treatment should be consulted with the attending physician.

During any procedure, the least invasive surgical techniques should be used and post-procedure hemostasis should be monitored.

Our study contains information relevant to daily practice, in situations of planned and emergency procedures, which are performed in patients treated chronically with anticoagulants.

The constant development of pharmacotherapy is associated with the introduction of new drugs to the market, including anticoagulants, so studies that present the issue described by us holistically are necessary.

The introduction of new anticoagulants into treatment may necessitate modification of the presented perioperative management with patients who will take them.

## Figures and Tables

**Table 1:** Antithrombotic drugs types.

Anticoagulant Drugs	Drug Group	Approximate Metabolism Time
Antiplatelet drugs	Acetylsalicylic acid	7 days
	P2Y12 receptor blockers (Clopidogrel, Prasugrel, Ticagrelor)	5 days
	Glycoprotein IIb/IIa inhibitors	36 h
	TxA2 synthetase inhibitors	no data
	prostacyclin PG12	2–4 h
Anticoagulants	Vitamin K antagonists (VKA)	3–5 days
	Non-vitamin K antagonists (NOACs)	1–2 days
	Heparins	1.5–24 h
	Pentasaccharides	15 h

**Table 2:** The table shows the management regimen for patients receiving double anticoagulant therapy.

Intraoperative Bleeding Risk	Risk of Thrombosis	
	Low Risk/Moderate Risk	High Risk
Low risk	Do not interrupt ASA or P2Y12 receptor blockers Treatment.	
Moderate risk	Do not interrupt ASA or P2Y12 receptor blockers Treatment, increase postoperative hemostasis.	
High risk	Do not interrupt ASA treatment, discontinue P2Y12 receptor blockers 5 days before the procedure after consultation with a cardiologist. Resume treatment 24–72 h postoperatively by administering a saturating dose of the discontinued drug.	Postpone scheduled procedures. In urgent cases do not interrupt ASA treatment, discontinue P2Y12 receptor blockers 5 days before the procedure after consultation with a cardiologist, resume treatment 24–72 h postoperatively by administering a saturating dose of the discontinued drug. Alternatively apply bringing therapy using Glycoprotein IIb/IIa inhibitors which should be discontinued 4 h before surgery.

**Table 3:** The table shows the management regimen for patients receiving Vitamin K antagonists (VKA).

Intraoperative Bleeding Risk	Risk of Thrombosis	
	Low Risk/Moderate Risk	High Risk
Low risk	Do not interrupt VKA treatment, 24 h before the procedure assess the INR value which should not exceed 3, if above the given value postpone the procedure until INR value normalizes.	
Moderate risk		
High risk	Decrease the perioperative INR value to 2.0–2.5.	Discontinue VKA treatment, Apply bridging therapy. In urgent cases administer FFP or prothrombin complex concentrate together with low doses of vitamin K (2.5–5.0 mg) intravenously or orally.

**Table 4:** The table shows the management regimen for patients taking NOACs, depending on renal function and bleeding risk.

Bleeding Risk	Renal Function	Perioperative Antithrombotic Therapy Management
Low to moderate risk	Normal kidney function or mild kidney function impairment—GFR ≥ 50 mL/min	Interrupt NOAC treatment 12–24 h before surgery, resume ≥6 h postoperatively
	Moderate to severe kidney function impairment—GFR 49–30 mL/min	Interrupt NOAC treatment 24–48 h before surgery
High risk	Normal renal function or mild renal function impairment—GFR ≥ 50 mL/min	Interrupt NOAC treatment 48 h before surgery, resume treatment 2–3 days post-surgery, consider a different antithrombotic therapy for 2–3 days
	Moderate to severe kidney function impairment–GFR 49–30 mL/min	Interrupt NOAC treatment 72 h before surgery

**Table 5:** The table shows the management regimen for patients on combination therapy: VKA/NOAC.

Intraoperative Bleeding Risk	Risk of Thrombosis	
	Low Risk/Moderate Risk	High Risk
Low risk	Discontinue NOAC treatment accordingly to the guidelines presented in Table 4. Do not interrupt VKA treatment, 24 h before the procedure assess the INR value which should not exceed 3, if above the given value postpone the procedure until INR value normalizes.	
Moderate risk		
High risk	Discontinue NOAC treatment accordingly to the guidelines presented in Table 4. Decrease the perioperative INR value to 2.0–2.5.	Discontinue NOAC treatment accordingly to the guidelines presented in Table 4. Discontinue VKA treatment, Apply bridging therapy. In urgent cases administer FFP or prothrombin complex concentrate together with low doses of vitamin K (2.5–5.0 mg) intravenously or orally.

**Table 6:** The table shows the management regimen for patients on combination therapy: VKA/ASA, VKA/Clopidogrel.

Intraoperative Bleeding Risk	Risk of Thrombosis	
	Low Risk/Moderate Risk	High Risk
Low risk	Do not interrupt ASA or P2Y12 receptor blockers treatment. Do not interrupt VKA treatment, 24 h before the procedure assess the INR value and proceed accordingly to the guidelines presented in Table 3.	
Moderate risk		
High risk	Consultation with the attending physician. Do not interrupt ASA or P2Y12 receptor blockers treatment.	
	24 h before the procedure assess the INR value and decrease the perioperative INR value to 2.0–2.5.	Discontinue VKA treatment, apply bridging therapy. In urgent cases proceed accordingly to the guidelines presented in Table 3.

## Data Availability

Not applicable.
